# Water Extract of *Agastache rugosa* Prevents Ovariectomy-Induced Bone Loss by Inhibiting Osteoclastogenesis

**DOI:** 10.3390/foods9091181

**Published:** 2020-08-26

**Authors:** Seon-A Jang, Youn-Hwan Hwang, Taesoo Kim, Hyun Yang, Jun Lee, Young Hye Seo, Jae-Il Park, Hyunil Ha

**Affiliations:** 1 Herbal Medicine Research Division, Korea Institute of Oriental Medicine, Yuseong-daero 1672, Yuseong-gu, Daejeon 34054, Korea; white7068@kiom.re.kr (S.-A.J.); hyhhwang@kiom.re.kr (Y.-H.H.); xotn91@kiom.re.kr (T.K.); hyunyang@kiom.re.kr (H.Y.); 2 Herbal Medicine Resources Research Center, Korea Institute of Oriental Medicine, Naju 58245, Korea; junlee@kiom.re.kr (J.L.); wnsl1118@kiom.re.kr (Y.H.S.); 3 Korea Basic Science Institute, Gwangju Center at Chonnam National University, Gwangju 61186, Korea; jaeil74@kbsi.re.kr

**Keywords:** *Agastache rugosa*, osteoclastogenesis, osteoporosis, ovariectomy

## Abstract

Estrogen deficiency in postmenopausal women causes homeostatic imbalance of bone, resulting in bone loss and osteoporosis. *Agastache rugosa*, a plant belonging to the Lamiaceae family, is an aromatic herb, and the leaves of this herb are widely used as food ingredients. Extracts of *A. rugosa* have various bioactivities including anti-HIV integration, anti-inflammatory, and anti-atherogenic properties. However, the beneficial effect of *A. rugosa* on bone has not been studied. Therefore, we investigated the effects of water extract of *A. rugosa* (WEAR) on osteoclast differentiation and estrogen deficiency-induced bone loss in ovariectomized (OVX) mice as an animal model for postmenopausal osteoporosis. The oral administration of WEAR remarkably improved OVX-induced trabecular bone loss and fat accumulation in the bone marrow. WEAR suppressed receptor activator of nuclear factor-κB ligand (RANKL)-induced osteoclast differentiation in osteoclast precursor cells, subsequently inhibiting resorption activity on a bone mimetic surface. WEAR inhibited the expression of cellular oncogene fos (c-Fos) and nuclear factor of activated T-cells cytoplasmic 1 (NFATc1), key osteoclastogenic transcription factors, by decreasing RANKL-induced activation of mitogen-activated protein kinases (MAPKs), and nuclear factor-κB (NF-κB) pathways. We also identified seventeen phytochemicals present in WEAR, including five phenols and twelve flavonoids, and found eleven bioactive constituents that have anti-osteoclastogenic effects. Collectively, these results suggest that WEAR could be used to treat and prevent postmenopausal osteoporosis by suppressing osteoclastogenesis.

## 1. Introduction

Osteoporosis is a metabolic disease characterized by low bone mass and microarchitectural deterioration of bone tissue: it can lead to impaired bone strength and an increased risk of fracture [1]. Bone remodeling is the continuous process through osteoclastic bone resorption and osteoblastic bone formation. This process is necessary to maintain mineral homeostasis and to repair damaged bone. However, excessive bone resorption by osteoclasts then bone formation by osteoblasts can cause pathological bone diseases, such as osteoporosis and rheumatoid arthritis [1,2].

Osteoclasts are multinucleated giant bone-resorbing cells formed by the proliferation, differentiation, and fusion of monocyte/macrophage lineage precursor cells. A receptor activator of nuclear factor-κB ligand (RANKL) is an essential cytokine that stimulates osteoclast differentiation, which includes proliferation, fusion, maturation, and resorption stages [3]. RANKL is produced by various bone cell types including mature osteoblasts, hypertrophic chondrocytes, and osteocytes. After RANKL binds to the RANK receptor on the cell surface of osteoclast precursors, the RANKL/RANK/TRAF6 axis activates multiple downstream signaling pathways, including nuclear factor-κB (NF-κB) and mitogen-activated protein kinases (MAPKs), which eventually lead to the activation of transcription factors needed for the differentiation, activation, and survival of osteoclasts [3]. These transcription factors include nuclear factor of activated T-cells cytoplasmic 1 (NFATc1) and cellular oncogene fos (c-Fos), which play crucial roles in osteoclastogenesis. Particularly, NFATc1 functions as a master transcription factor for osteoclast differentiation, and its induction is dependent on c-Fos [4,5]. Moreover, NFATc1 up-regulates the expression of osteoclast-specific genes, such as Atp6v0d2, dendritic cell–specific transmembrane protein (DC-STAMP), c-Src, cathepsin-K, and tartrate-resistant acid phosphatase (TRAP) [6]. A schematic diagram of the RANK-RANKL signaling in osteoclastogenesis is shown in Supplementary Figure S1.

*Agastache rugosa* (Fisch. & C.A. Mey.) Kuntze (*A. rugosa*), the Korean mint, also known as Indian mint, wrinkled giant hyssop, blue licorice, and huo xiang, is mainly distributed throughout East Asia. The leaves of *A. rugosa* are widely used as food ingredients, especially as a spice in various soups in Korea. Water extract of *A. rugosa* has traditionally been used to treat several symptoms, including vomiting, fever, headaches, diarrhea, and halitosis. Chemical studies have shown that the chemical composition of *A. rugosa* mainly includes flavonoids, terpenoids, and essential oils [7,8]. Previous studies reported that extracts of *A. rugosa* have various bioactivities, including anti-HIV integration, anti-inflammatory, anti-tumor, anti-fungal, anti-oxidant, and anti-atherogenic properties [9,10]. However, the anti-osteoporotic effect of water extract of *A. rugosa* (WEAR) has not been studied. In the present study, we investigated the beneficial effects of WEAR on estrogen-deficiency-induced osteoporosis using ovariectomized (OVX) mice and its inhibitory action on osteoclast differentiation.

## 2. Materials and Methods

### 2.1. Materials

Antibodies against phospho-Jun N-terminal kinase (p-JNK), JNK, phospho-p38, p38, phosphor-extracellular signal-regulated kinase (p-ERK), ERK, IκBα, and β-actin were obtained from Cell Signaling Technology (Danvers, MA, USA). Antibodies against c-Fos and NFATc1 were purchased from Santa Cruz Biotechnology (Santa Cruz, CA, USA). Recombinant macrophage colony-stimulating factor (M-CSF) and RANKL were obtained as previously described [11].

### 2.2. Preparation of WEAR

The shade-dried aerial parts (leaves, stems, and flowers) of *A. rugose,* cultivated in Gyeongsangbuk-do province, Korea, were obtained from the National Development Institute of Korean Medicine (Gyeongsan, Korea), and a voucher specimen (number #134) was deposited at the herbarium of the Korean Institute of Oriental Medicine. The dried aerial parts of *A. rugosa* (0.5 kg) were extracted with distilled water (3.5 L) at the boiling point under reflux for 3 h and lyophilized after filtration to obtain 35 g of lyophilized powder (yield: 7.0%). The lyophilized powder dissolved in distilled water to prepare WEAR.

### 2.3. Animal Experiment and Bone Analysis

Female C57BL/6J Jms Slc mice (6 weeks old, 18–20 g) were purchased from Japan SLC Inc. (Shizuoka, Japan) and housed under standard conditions (22 °C ± 2 °C and 55 ± 5% humidity under a 12 h light/dark cycle). The mice were provided filtered tap water and commercial pelleted food ad libitum. All experimental protocols (approval number: 19-KE-216) were approved by the Institutional Animal Care and Use Committee at Knotus (Guri, Korea). The mice were either sham-operated or OVX via bilateral dorsal incisions under Zoletil (Virbac, Carros, France) and Rumpun (Bayer, Leverkusen, Germany) anesthesia, according to a previously reported method [12]. The mice were randomly divided into four groups (*n* = 6): (1) sham (normal control), (2) OVX (negative control), (3) OVX fed WEAR 100 mg/kg/day (WEAR-L), and (4) OVX fed WEAR 300 mg/kg/day (WEAR-H). The mice received a normal-fat diet (10 kcal%; Research Diet, New Brunswick, NJ, USA) together with WEAR or vehicle by oral administration once daily for 5 weeks. For histological analysis, the femurs were fixed using 4% formaldehyde, decalcified with RDO Gold (RDO, Aurora, IL, USA), embedded in paraffin, and then sectioned at 5 μm thickness. The sections were stained with hematoxylin and eosin (H&E) and photographed. Images were analyzed using ImageJ software. The microarchitecture of the distal femur was analyzed by using a micro-computed tomography (µ-CT) imaging system (SkySacn 1276, Bruker, Kontich, Belgium). The raw images were reconstructed and analyzed with SkySacn NRecon (Version 1.7.4.2, Bruker, Kontich, Belgium) and CTAn software (Version 1.20.3.0, Bruker, Kontich, Belgium), respectively. Trabecular bone mineral density (BMD), trabecular number (Tb.N), bone volume per tissue volume (BV/TV), trabecular separation (Tb.Sp), and trabecular thickness (Tb.Th) were calculated.

### 2.4. Cell Viability Assay

Mouse bone marrow-derived macrophages (BMMs) were prepared from mouse bone marrow cells, as previously described [13]. The cells (2 × 10^4^ cells/well) were treated with or without WEAR (33.3, 100, and 200 μg/mL) in the presence of M-CSF (60 ng/mL) for 24 h. Cell viability assessed by the Cell Counting Kit-8 assay (Dojindo Molecular Technologies Inc., Rockville, MD, USA) in a 96-well plate.

### 2.5. Tartrate-Resistant Acid Phosphatase (TRAP) Assay and Resorption Assay

BMMs were cultured in α-MEM medium containing M-CSF (60 ng/mL), 1% penicillin/streptomycin, and 10% fetal bovine serum (FBS). BMMs (1 × 10^4^ cells/well) were treated with or without WEAR (33.3, 100, and 200 μg/mL) or phytochemicals (5, 10, and 20 μM) as indicated in the presence of M-CSF (60 ng/mL) and RANKL (50 ng/mL) for 4 days in a 96-well plate. The cells were fixed using 10% neutral buffered formalin, permeabilized using 0.1% Triton X-100, and then incubated with TRAP buffer (50 mM sodium tartrate and 0.12 M sodium acetate, pH 5.2) containing p-nitrophenyl phosphate for 20 min at 37 °C. The enzymatic reaction was stopped by adding 0.1 M NaOH and measured at 405 nm. The cells were also stained with TRAP buffer contained fast red violet LB salt and naphthol AS-MX phosphate and photographed using an optical microscope. To assess bone resorption activity, BMMs were differentiated into osteoclasts on an Osteo Assay Surface plate (Corning, New York, NY, USA) in the presence of M-CSF and RANKL with or without WEAR (33.3, 100, and 200 μg/mL) for 4 days. Resorption pits were observed using a microscope after removing cells with 5% sodium hypochlorite [14].

### 2.6. Quantitative Real-Time Polymerase Chain Reaction (qPCR) Analysis

Total RNA was isolated using an RNA-spin total RNA Extraction Kit (iNtRON Biotechnology Inc., Sungnam, Korea), and RNA concentration was measured with NanoDrop spectrophotometer (ThermoFisher Scientific, Pittsburgh, PA, USA). Two micrograms of total RNA were used for cDNA synthesis using High-Capacity cDNA Reverse Transcription Kit (Thermo Fisher Scientific, Waltham, MA, USA). The qPCR was performed using the TaqMan primers for NFATc1 (Mm00479445_m1), c-Fos (Mm00487425_m1), Atp6v0d2 (Mm00656638_m1), DC-STAMP (Mm01168058_m1), c-Src (Mm00436785_m1), and 18S rRNA (Hs99999901_s1) and the TaqMan Universal Master Mix Ⅱ (Applied Biosystems, Foster City, CA, USA) on an ABI 7500 Real-Time PCR system (Applied Biosystems, Foster City, CA, USA). The PCR cycles were performed as an initial denaturation step (95 °C for 10 min) and 40 amplification cycles (95 °C for 15 s and at 60 °C for 1 min). The relative quantification of the target genes was calculated using the ΔΔCt method, normalized to an endogenous control (18S rRNA), and expressed as fold change relative to untreated controls.

### 2.7. Western Blot Analysis

Western blot was performed as previously described [15]. Briefly, the cells were washed twice with cold PBS and lysed in PRO-PREP^TM^ Protein Extraction Solution (iNtRON Biotechnology, Sungnam, Korea) containing protease and phosphatase inhibitor. Cell lysates were cleared by centrifugation at 13,000× *g* for 30 min at 4 °C. Protein concentrations were determined by the BCA Protein Assay Kit (Bio-Rad Laboratories, Hercules, CA, USA). Protein samples containing equal amounts of total protein were electrophoresed using 10% sodium dodecyl sulfate-polyacrylamide gel electrophoresis (SDS-PAGE) and transferred to a polyvinylidene fluoride membrane. The membranes were blocked with 5% skim milk in Tris-buffered saline with 0.1% Tween 20 (TBST) at room temperature for 1 h, incubated with primary antibodies against c-Fos, NFATc1, p-p38, p38, p-JNK, JNK, p-ERK, ERK, IκBα, and β-actin (1:1000 dilution) overnight at 4 °C, and then washed with TBST six times for 5 min each. The membranes were washed and then incubated with horseradish peroxidase-conjugated secondary antibodies (1:5000 dilution) at room temperature for 1 h and washed with TBST six times. The target proteins were detected with Pierce ECL Western Blotting Substrate and visualized with the ChemiDoc Imaging System (Bio-Rad, Hercules, CA, USA).

### 2.8. Ultrahigh-Performance Liquid Chromatography and Tandem Mass Spectrometry (UHPLC–MS/MS) Analysis

Seventeen reference standards with more than 98% were used to identify the phytochemicals in WEAR. Chlorogenic acid and neochlorogenic acid were purchased from ChemFace (Wuhan, China), and rosmarinic acid, citrusin C, nepetoidin B, diosmetin-7-*O-β*-d-glucopyranoside, tilianin, isoagastachoside, acacetin, apigetrin, phlorizin, luteolin, diosmetin, apigenin, acacetin-7-*O*-(6″-*O*-malonyl)-β-d-glucopyranoside, acacetin-7-*O*-(3″-*O*-acetyl)-β-d-glucopyranoside, and acacetin-7-*O*-(2″-*O*-acetyl-6″-*O*-malonyl)-β-d-glucopyranoside were isolated from *A. rugosa* as previously described [7]. The Dionex UltiMate 3000 system equipped with a Thermo Q-Exactive mass spectrometer (Thermo Fisher Scientific, San Jose, CA, USA) was used as in previously described methods [16]. Xcalibur software (Version 3.0, Thermo Fisher Scientific, Foster, CA, USA) was used for data acquisition and analysis.

### 2.9. Statistical Analysis

Data are presented as mean ± standard deviation (SD) for in vitro studies and as mean ± standard error of the mean (SEM) for in vivo studies. Statistical comparisons were performed using a one-way analysis of variance (ANOVA) and Dunnett’s post hoc test or a two-way ANOVA and Bonferroni’s post hoc test using Prism software (Graphpad, San Diego, CA, USA). A level of *p* < 0.05 was considered statistically significant compared to the control.

## 3. Results and Discussion

### 3.1. WEAR Attenuates OVX-Induced Bone Loss

**Figure 1:**
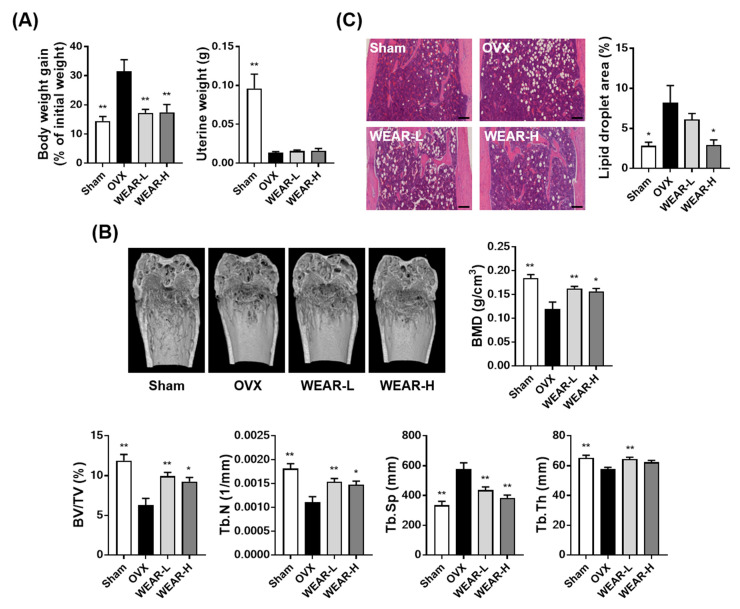
Effects of water extract of *A. rugosa* (WEAR) on bone loss in ovariectomized (OVX) mice. Sham or OVX mice were treated with vehicle or WEAR for 5 weeks. (**A**) Alteration of body weight gain and uterine weight. (**B**) µ-CT images, trabecular bone mineral density (BMD), and bone morphometric analysis of the trabecular bone. (**C**) Histopathological images of the distal femur by hematoxylin and eosin (H&E) staining (scale bar, 100 µm) and the fat area in the bone marrow. OVX, ovariectomized mice; WEAR-L, OVX mice fed WEAR 100 mg/kg/day; WEAR-H, OVX mice fed WEAR 300 mg/kg/day; BMD, trabecular bone mineral density; BV/TV, bone volume per tissue volume; Tb.N, trabecular number; Tb.Sp, trabecular separation; Tb.Th, trabecular thickness. * *p* < 0.05, ** *p* < 0.01 vs. the OVX group.

To investigate the beneficial effects of WEAR on bone loss, we used an OVX mouse model. OVX-induced estrogen deficiency leads to a reduction in BMD with bone metabolic changes similar to those occurring in human postmenopausal osteoporosis [17]. OVX also leads to weight gain and uterine atrophy due to estrogen deficiency [18]. As shown in Figure 1A, the OVX group showed a significant increase in body weight and decrease in uterine weight at 5 weeks after the operation. WEAR administration (100 and 300 mg/kg) markedly suppressed OVX-induced body weight gain but did not affect uterine atrophy. The morphological examination of the bone microstructure under pathophysiological conditions can provide critical information concerning the degree of bone loss. The morphological alterations in osteoporotic animal model are well known to occur in cortical and trabecular bone, and morphometric indices for bone mass and bone formation decrease with increasing bone resorption [19]. The morphometric analysis by μ-CT is defined bone quality using calculated parameters, such as bone thickness, separation, and density. In general, the circumstantial evaluation of trabecular microstructure uses BV/TV, Tb.N, Tb.Sp, and Tb.Th [20]. Estrogen deficiency has been shown to mainly alter trabecular bone architecture unlike senile (age-related) osteoporosis [21]. It has been also reported that bone marrow adipocyte accumulation damages bone healing and regeneration [22]. Thus, to investigate the bone protective effects of WEAR on OVX mice, bone microstructure in the distal femoral trabecular bone and bone marrow fat accumulation were examined. OVX markedly induced trabecular bone loss in the distal femoral metaphysis with an inhibition in BMD, BV/TV, Tb.N, and Tb.Th and an increase in Tb.Sp, which was significantly suppressed by WEAR administration (Figure 1B). Similarly, OVX resulted in fat accumulation in the bone marrow, which was decreased by WEAR-H administration (Figure 1C). Since WEAR did not affect OVX-induced uterine atrophy, it is likely that the beneficial effects of WEAR on bone are independent of its phytoestrogen property. These results demonstrate that WEAR can alleviate OVX-induced bone loss and fat accumulation, suggesting that WEAR might be a promising candidate for the treatment of postmenopausal osteoporosis.

### 3.2. WEAR Inhibits RANKL-Induced Osteoclast Differentiation

**Figure 2:**
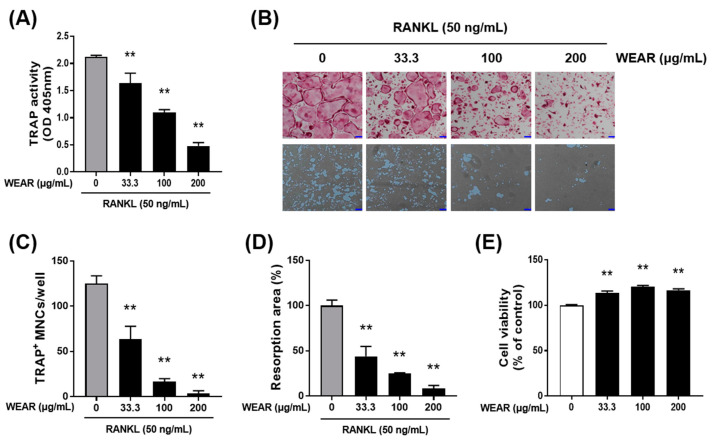
Effects of WEAR on receptor activator of nuclear factor-κB ligand (RANKL)-induced osteoclast differentiation. (**A**–**D**) Bone marrow-derived macrophages (BMMs) were cultured in a tissue culture plate (A, B upper panel, and C) or in an Osteo Assay Surface plate (B lower panel and D) with or without WEAR (33.3, 100, and 200 μg/mL) in the presence of macrophage colony-stimulating (M-CSF) (60 ng/mL) and RANKL (50 ng/mL) for 4 days. (**A**) The total cellular tartrate-resistant acid phosphatase (TRAP) activity. (**B**) Representative microscopic images of TRAP staining (upper panel; scale bar, 100 µm) and resorption pits (lower panel; scale bar, 100 µm). (**C**) The number of pink-colored TRAP-positive multinucleated cells (MNCs) with more than three nuclei. (**D**) Relative resorption area. (**E**) Cell viability was assessed in BMMs treated with or without WEAR for 24 h. * *p* < 0.05, ** *p* < 0.01 vs. control.

Because WEAR had a beneficial effect on bone loss in vivo, we next examined the effects of WEAR on osteoclasts, which are predominantly responsible for bone loss. Osteoclastogenesis is dependent on M-CSF and RANKL. M-CSF is known to promote the proliferation and survival of osteoclast precursors, and RANKL directly induces the differentiation of osteoclast [23]. We investigated whether WEAR suppresses RANKL-induced osteoclast differentiation and resorption activity. TRAP activity is commonly used as an indicator for osteoclast differentiation. WEAR decreased RANKL-induced TRAP activity (Figure 2A) and osteoclast formation (Figure 2B upper panel and C) in BMMs in a dose-dependent manner. The specific ability of osteoclasts relates to bone absorption. When attached to bone matrix, osteoclasts polarize their membrane and secret hydrochloric acid and acidic proteases that degrade the bone matrix [1]. When BMMs were differentiated into osteoclasts on a bone mimetic surface, numerus resorption pits were observed, and treatment with WEAR dose-dependently decreased the area of resorption pits (Figure 2B lower panel and D). WEAR slightly increased the cell viability of BMMs at the tested concentrations (Figure 2E), suggesting that the inhibitory effect of WEAR is not due to cytotoxicity.

### 3.3. WEAR Inhibits RANKL-Induced Early Signaling Pathways

**Figure 3:**
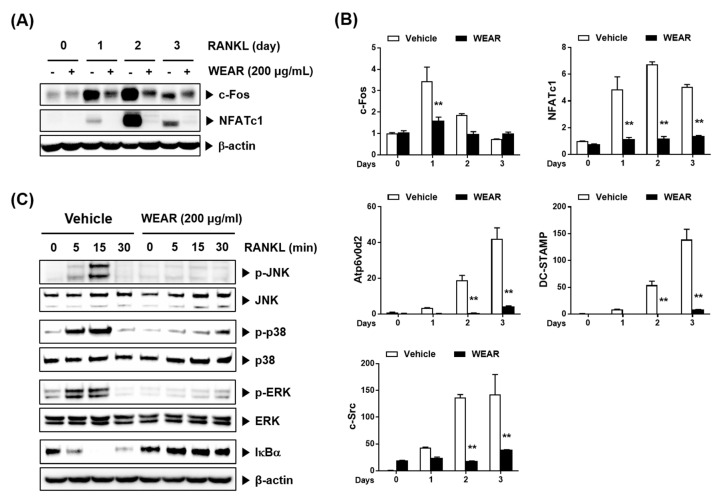
Effects of WEAR on RANKL-induced signaling pathways in BMMs. BMMs were treated with or without WEAR (200 μg/mL) and RANKL (50 ng/mL) for the indicated days. (**A**) Western blot analysis of c-Fos and NFATc1. (**B**) Relative mRNA expression of the indicated genes by real-time polymerase chain reaction (PCR). ** *p* < 0.01 vs. vehicle. (**C**) BMMs pretreated with WEAR (200 μg/mL) for 3 h were stimulated with RANKL for the indicated times. The indicated proteins were detected by Western blot.

NFATc1 is a master transcription factor involved in RANKL-induced osteoclastogenesis [5]. c-Fos is also indispensable for RANKL-induced osteoclastogenesis and functions as a crucial upstream activator of NFATc1 [4]. Because WEAR suppressed RANKL-induced osteoclast differentiation, we investigated the effect of WEAR on the expression of c-Fos and NFATc1. WEAR strongly attenuated the mRNA and protein expression of c-Fos and NFATc1 during RANKL-induced osteoclast differentiation (Figure 3A,B). NFATc1 is known to regulate osteoclast-specific genes engaged in osteoclast fusion, maturation, and resorption including the Atp6v0d2, DC-STAMP, and c-Src [6], so we next investigated the expression of these genes. Atp6v0d2 and DC-STAMP are essential molecules for the fusion of mononuclear osteoclasts [24,25], and c-Src is required for osteoclastic bone resorption [26]. RANKL significantly induced the expression of these osteoclast-specific genes at the mRNA level, while WEAR dramatically inhibited the expression of these genes (Figure 3B). To further investigate WEAR-induced suppression of c-Fos and NFATc1, we examined the effects of WEAR on RANKL-induced early signaling pathways. RANK stimulation by RANKL initially activates both the MAPKs (p38, ERK, and JNK) and NF-κB signaling pathways, which are involved in the induction of c-Fos and NFATc1 [27,28,29,30]. RANKL stimulated the activation of JNK, ERK, and p38, assessed by their phosphorylation status, and the classical NF-κB pathway, assessed by the degradation of IκBα in BMMs, which was markedly suppressed by WEAR (Figure 3C). Collectively, our results suggest that the inhibitory effect of WEAR on osteoclast differentiation can be attributed to the inhibition of the early RANK signaling pathways and subsequent downregulation of c-Fos, NFATc1, and osteoclast-specific genes. The proposed inhibitory mechanisms are supported by previous studies [31,32].

### 3.4. The Phytochemical Profile of WEAR

**Figure 4:**
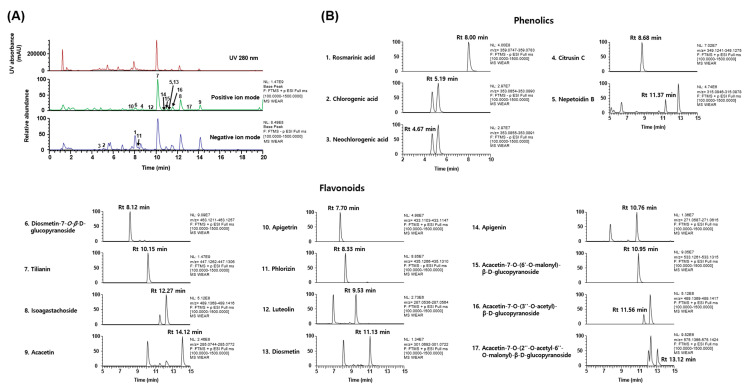

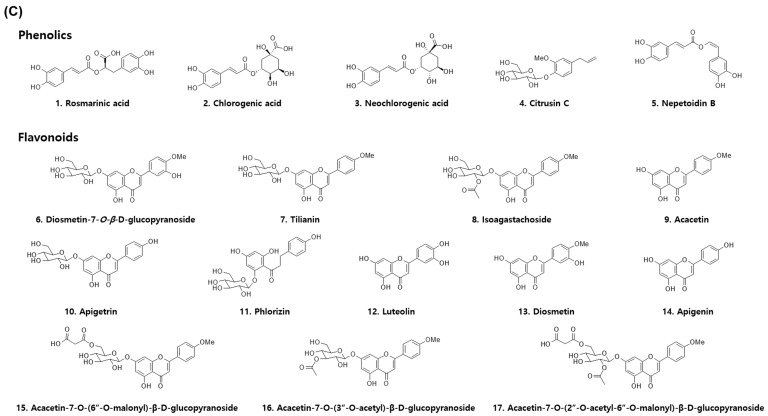
The phytochemical profile of WEAR assessed by UHPLC–MS/MS analysis. (**A**) UV and base peak chromatograms of WEAR. (**B**) Extracted ion chromatograms and (**C**) chemical structures of the seventeen phytochemicals identified in WEAR. Peak numbers correspond to the phytochemicals listed in Table 1. Rt, retention time.

To clarify the biological properties of WEAR and the mechanisms underlying its benefits, we next investigated the phytochemical profile of WEAR. *A. rugosa* has been reported to contain essential oils and several types of compounds, such as phenylpropanoids, lignans, flavonoids, and terpenoids [7,8]. The typical UV (at 280 nm) base peak chromatograms and the extracted ion chromatogram for each WEAR analyte are shown in Figure 4. UHPLC–MS/MS analysis of WEAR identified five phenolics (i.e., rosmarinic acid, chlorogenic acid, neochlorogenic acid, citrusin C, and nepetoidin B) and twelve flavonoids (diosmetin-7-*O-β*-d-glucopyranoside, tilianin, isoagastachoside, acacetin, apigetrin, phlorizin, luteolin, diosmetin, apigenin, acacetin-7-*O*-(6″-*O*-malonyl)-β-d-glucopyranoside, acacetin-7-*O*-(3″-*O*-acetyl)-β-d-glucopyranoside, and acacetin-7-*O*-(2″-*O*-acetyl-6″-*O*-malonyl)-β-d-glucopyranoside) by comparing retention times of the authentic standards and the mass spectra (Table 1).

### 3.5. Phytochemicals of WEAR Inhibits RANKL-Induced Osteoclast Differentiation

**Figure 5:**
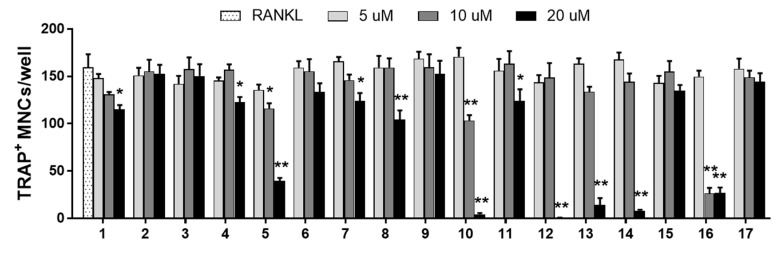
Effects of the phytochemicals in WEAR on osteoclast differentiation in BMMs. BMMs were cultured with or without the seventeen phytochemicals (5, 10, and 20 μM) in the presence of RANKL for 4 days. Cells were stained for TRAP activity, and the number of TRAP-positive multinucleated cells (≥ three nuclei) was counted. 1. Rosmarinic acid, 2. Chlorogenic acid, 3. Neochlorogenic acid, 4. Citrusin C, 5. Nepetoidin B, 6. Diosmetin-7-*O-β*-d-glucopyranoside, 7. Tilianin, 8. Isoagastachoside, 9. Acacetin, 10. Apigetrin, 11. Phlorizin, 12. Luteolin, 13. Diosmetin, 14. Apigenin, 15. Acacetin-7-*O*-(6″-*O*-malonyl)-β-d-glucopyranoside, 16. Acacetin-7-*O*-(3″-*O*-acetyl)-β-d-glucopyranoside, 17. Acacetin-7-*O*-(2″-*O*-acetyl-6″-*O*-malonyl)-β-d-glucopyranoside. * *p* < 0.05, ** *p* < 0.01 vs. RANKL.

In order to determine whether the inhibitory effect of WEAR on osteoclastogensis may result from the bioactive properties of the above-mentioned phytochemicals, we evaluated the effects of these phytochemicals on osteoclast differentiation. As a result, eleven bioactive compounds (rosmarinic acid, citrusin C, nepetoidin B, tilianin, isoagastachoside, apigetrin, phlorizin, luteolin, diosmetin, apigenin, and acacetin-7-*O*-(3”-*O*-acetyl)-β-d-glucopyranoside) inhibited RANKL-induced osteoclast differentiation (Figure 5). Similar to our results, previous studies have shown that rosmarinic acid inhibits RANKL-induced bone loss via suppression of p38-mediated osteoclast differentiation [44], and diosmetin decreases LPS-induced osteolysis via inhibition of ERK, JNK, and reactive oxygen species-mediated osteoclast formation and differentiation [45]. In addition, phlorizin, luteolin, and apigenin prevent OVX-induced postmenopausal bone loss [46,47,48]. In the present study, we newly identified six bioactive compounds (citrusin C, nepetoidin B, tilianin, isoagastachoside, apigetrin, and acacetin-7-*O*-(3”-*O*-acetyl)-β-d-glucopyranoside) in WEAR that have anti-osteoclastogenic activities, although their underlying mechanisms remain to be elucidated. Thus, these results suggest that the anti-osteoclastogenic effect of WEAR may result from the complementary effects of these bioactive constituents.

## 4. Conclusions

In conclusion, this is the first study demonstrating the anti-osteoclastogenic and anti-osteoporotic effects of WEAR. WEAR reduced OVX-induced bone loss and fat accumulation in the bone marrow in vivo. WEAR suppressed osteoclastogenesis by inhibiting the expression of c-Fos and NFATc1 by interfering with the early RANK signal pathways in osteoclast precursors. We also identified eleven bioactive constituents in WEAR that have anti-osteoclastogenic activities. These findings suggest that WEAR may be useful as functional food or nutraceutical for the treatment of menopausal osteoporosis.

## Figures and Tables

**Table 1:** The phytochemical profile of WEAR analyzed by ultrahigh-performance liquid chromatography and tandem mass spectrometry UHPLC-MS/MS.

No	Rt (min)	Calculated (m/z)	Estimated (m/z)	Adducts	Error (ppm)	Formula	MS/MS Fragments (m/z)	Identifications [References]
**Phenolics**								
1	8.00	359.0772	359.0765	[M-H]^-^	−1.9548	C_18_H_16_O_8_	135.0435, 161.0228, 179.0337, 197.0443	Rosmarinic acid [7,33]
2	5.19	353.0878	353.0872	[M-H]^-^	−1.8460	C_16_H_18_O_9_	93.0326, 173.0439, 191.0547	Chlorogenic acid [34,35]
3	4.67	353.0878	353.0873	[M-H]^-^	−1.5003	C_16_H_18_O_9_	135.0437, 173.0438, 179.0334, 191.0547	Neochlorogenic acid [34,35]
4	8.68	349.1258	349.1258	[M+Na]^+^	0.0672	C_16_H_22_O_7_	349.1258	Citrusin C [7,36]
5	11.37	315.0863	315.0862	[M+H]^+^	−0.2137	C_17_H_14_O_6_	163.0390	Nepetoidin B [7,37]
**Flavonoids**								
6	8.12	463.1235	463.1234	[M+H]^+^	−0.1661	C_22_H_22_O_11_	286.0473, 301.0707	Diosmetin-7-*O-β*-d-glucopyranoside [7,38]
7	10.15	447.1286	447.1284	[M+H]^+^	−0.3400	C_22_H_22_O_10_	285.0756	Tilianin [7,39]
8	12.27	489.1391	489.1392	[M+H]^+^	0.0412	C_24_H_24_O_11_	285.0755	Isoagastachoside [7]
9	14.12	285.0758	285.0758	[M+H]^+^	0.0882	C_16_H_12_O_5_	242.0584, 285.0756	Acacetin [7,40]
10	7.70	433.1129	433.1125	[M+H]^+^	−0.8570	C_21_H_20_O_10_	271.0599	Apigetrin [7,41]
11	8.33	435.1297	435.1288	[M-H]^-^	−2.1059	C_21_H_24_O_10_	167.0333, 179.0334, 273.0761, 297.0728	Phlorizin [7,42]
12	9.53	287.0550	287.0550	[M+H]^+^	−0.1667	C_15_H_10_O_6_	287.0550	Luteolin [7,43]
13	11.13	301.0707	301.0707	[M+H]^+^	0.0621	C_16_H_12_O_6_	286.0474	Diosmetin [7,38]
14	10.76	271.0601	271.0601	[M+H]^+^	−0.0402	C_15_H_10_O_5_	271.0599	Apigenin [7,39]
15	10.95	533.1290	533.1288	[M+H]^+^	−0.2341	C_25_H_24_O_13_	285.0757	Acacetin-7-*O*-(6″-*O*-malonyl)-β-d-glucopyranoside [7]
16	11.56	489.1391	489.1393	[M+H]^+^	0.2284	C_24_H_24_O_11_	285.0756	Acacetin-7-*O*-(3″-*O*-acetyl)-β-d-glucopyranoside [7]
17	13.12	575.1395	575.1395	[M+H]^+^	−0.0063	C_27_H_26_O_14_	285.0756	Acacetin-7-*O*-(2″-*O*-acetyl-6″-*O*-malonyl)-β-d-glucopyranoside [7]

## Supplementary Materials

The following are available online at https://www.mdpi.com/2304-8158/9/9/1181/s1, Figure S1: The RANK-RANKL signaling in osteoclastogenesis.

## Abbreviations

BMM	bone marrow-derived macrophage
M-CSF	macrophage colony-stimulating factor
OVX	ovariectomized
RANKL	receptor activator of nuclear kappa-B ligand
WEAR	water extract of *A. rugosa*
