# Supplementary figures and images for: Water Extract of Agastache rugosa Prevents Ovariectomy-Induced Bone Loss by Inhibiting Osteoclastogenesis
Source: Foods. 2020 Aug 26;9(9):1181. doi: 10.3390/foods9091181 (PMC7555585; doi:10.3390/foods9091181)

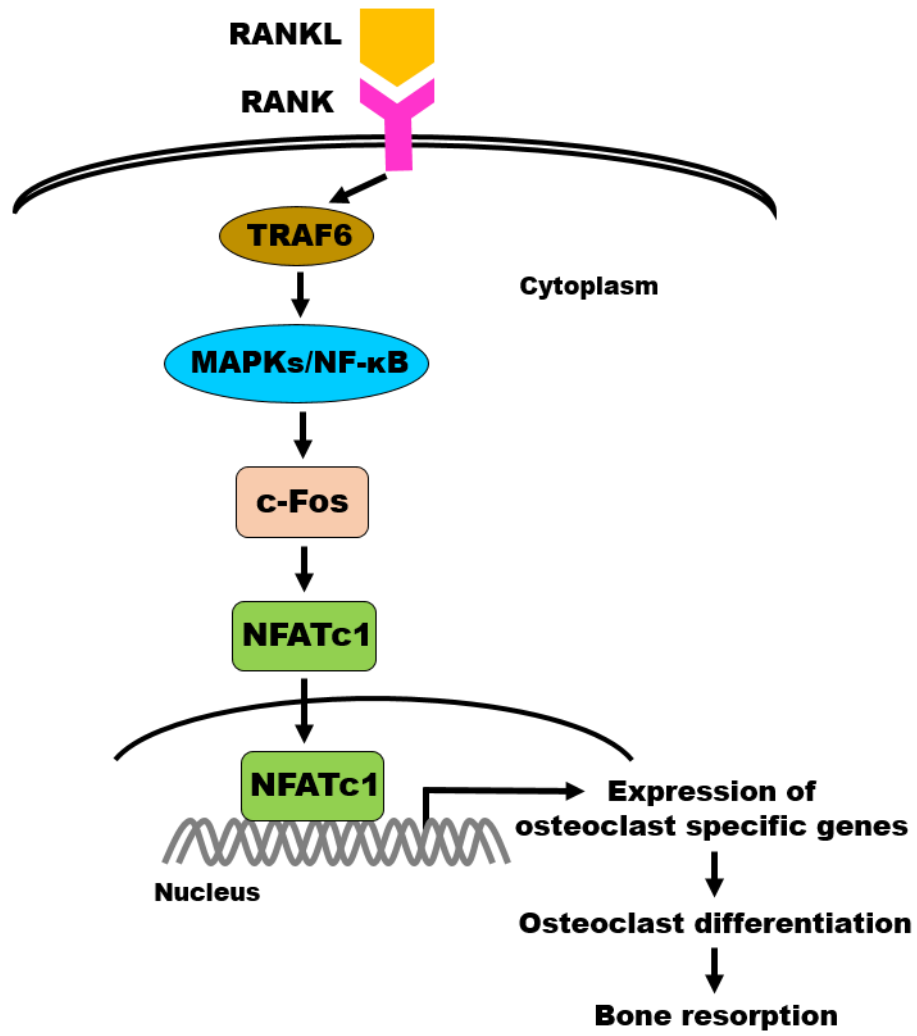

**Figure 1.** The RANK-RANKL signaling in osteoclastogenesis.

Supplement: Supplementary file 1 [file foods-09-01181-s001.pdf]
